# Progress in biological age research

**DOI:** 10.3389/fpubh.2023.1074274

**Published:** 2023-04-12

**Authors:** Zhe Li, Weiguang Zhang, Yuting Duan, Yue Niu, Yizhi Chen, Xiaomin Liu, Zheyi Dong, Ying Zheng, Xizhao Chen, Zhe Feng, Yong Wang, Delong Zhao, Xuefeng Sun, Guangyan Cai, Hongwei Jiang, Xiangmei Chen

**Affiliations:** ^1^The First Affiliated Hospital, and College of Clinical Medicine of Henan University of Science and Technology, Luoyang, China; ^2^Department of Nephrology, First Medical Center of Chinese PLA General Hospital, Nephrology Institute of the Chinese People's Liberation Army, State Key Laboratory of Kidney Diseases, National Clinical Research Center for Kidney Diseases, Beijing Key Laboratory of Kidney Disease Research, Beijing, China; ^3^Department of Nephrology, Hainan Hospital of Chinese PLA General Hospital, Hainan Academician Team Innovation Center, Sanya, China

**Keywords:** aging, biological age, aging biomarkers, chronological age, deep learning, age

## Abstract

Biological age (BA) is a common model to evaluate the function of aging individuals as it may provide a more accurate measure of the extent of human aging than chronological age (CA). Biological age is influenced by the used biomarkers and standards in selected aging biomarkers and the statistical method to construct BA. Traditional used BA estimation approaches include multiple linear regression (MLR), principal component analysis (PCA), Klemera and Doubal’s method (KDM), and, in recent years, deep learning methods. This review summarizes the markers for each organ/system used to construct biological age and published literature using methods in BA research. Future research needs to explore the new aging markers and the standard in select markers and new methods in building BA models.

## Introduction

Aging is accompanied by a progressive decline in physiological functions and an accumulation of damage to the body, leading to an increased risk of morbidity and mortality (1). Based on birth date, chronological age (CA) is the traditional criterion for assessing aging. However, the degree of aging may vary significantly between individuals with the same CA (2). Therefore, CA is not the best indicator for evaluating the degree of aging in human individuals.

To seek a better index to assess the degree of aging of individuals, biological age (BA) (3, 4) are used as alternatives to CA to estimate aging status. BA is the most popularly used model. Aging markers are the basis for constructing biological age, in this article we summarize the markers used in constructing biological age. There are many ways to classify markers of aging, e.g., the aging markers can classify into two categories: histology-based data (DNA methylation, metabolomics, proteomics, etc.), and clinical biomarkers obtained from blood chemistry, hematology, anthropometry, and organ function test measurements (5, 6). The “aging clock” developed from omics data is another form of biological age, multiple omics data can be combined to build the clock (7). Until now, omics data have rarely been used in the construction of BA because of the high cost of its application in large-scale populations. Previously built BA models commonly choose aging biomarkers in multiple organs/systems, such as blood biomarkers (8, 9), genetic indicators (10), and physical activity data (11, 12). Biomarkers from diverse organs are more reflective of the overall body state. To build the BA model, these biomarkers apply different model building methods like multiple linear regression (MLR) (13, 14), principal component analysis (PCA) (15, 16), Klemera and Doubal’s method (KDM) (3, 17), deep learning (8, 12), and other methods.

Previous studies have focused on the construction of BA models using different methods, but fewer studies have compared the BA models constructed by these methods, especially the advantages and disadvantages between deep learning and traditional methods. This review focuses on BA models constructed by four common approaches, namely composite or combined biomarkers that include a lot of aging markers. A more accurate biological age can only be constructed on the basis of knowing the advantages and disadvantages of existing methods. Table 1 provides a review of the more important, influential, and newly published literature with the four approaches above (3, 4, 8–16, 18–51), including cross-sectional and longitudinal studies.

**Table 1:** The basic information of BA models in different populations.

Assessment methods	Researchers	Year	Country	Sample size	Age range	Population	Aging biomarkers (Candidate → Final)
MLR	Hollingsworth et al.	1965	Japan	169 Males 268 Females	10–70+ years	General population	17 → 9
MLR	Webster and Logie	1976	Australia	1,080 Females	21–83 years	General population	37 → 7
MLR	Takeda et al.	1982	Japan	200 Males	20–69 years	Healthy population	10 → 5
MLR	Voitenko and Tokar	1983	Soviet Union	88 Males 109 Females	19–73 years	General population	122 → 11
MLR	Dubina et al.	1984	Soviet Union	100 Males 63 Females	60–100 years	Healthy population	21 → 3
MLR /PCA	Nakamura et al.	1988	Japan	462 Males	30–80 years	Healthy population	30 → 11
PCA	Nakamura et al.	1989	Japan	69 Males	Average 42.6 ± 9.4 years	Healthy population	18 → 7
PCA	Nakamura et al.	1990	Japan	65 Females	20–64 years	Healthy population	18 → 9
PCA	Nakamura et al.	1996	Japan	221 Males	20–85 years	Healthy population	17 → 8
PCA	Nakamura and Miyao	2003	Japan	86 Males	31–77 years	Healthy population (including some early functional decline or disease)	25 → 9
PCA	Ueno et al.	2003	Japan	981 Females (cross-sectional study) 110 Females (longitudinal study)	28–80 years	Healthy population	31 → 5
PCA	Nakamura and Miyao	2007	Japan	86 Males	31–77 years	Healthy population (including some early functional decline or disease)	29 → 5
MLR	Bae et al.	2008	Korea	1,302 Males 2,273 Females	40–88 years	General population	80 → 25
PCA	Nakamura and Miyao	2008	Japan	86 Males 93 Females	31–77 years	Healthy population (including some early functional decline or disease)	29 → 5
PCA	Park et al.	2009	Korea	1,588 Males	30–77 years	Healthy population (including some early functional decline or disease)	11
PCA	Bai et al.	2010	China	392 Males 460 Females	30–98 years	Healthy population (including some early functional decline or disease)	108 → 8
MLR/PCA/KDM	Cho et al.	2010	Korea	200 Males	30–70 years	General population	16 → 11/3 principal components
PCA	Jee et al.	2012	Korea	1,604 Males 760 Females	30–85 years	Healthy population	14 → 8
MLR	Bae et al.	2013	Korea	66,168 Males 55,021 Females	20–89 years	General population	34
MLR/PCA/KDM	Levine	2013	United States	9,389 People	30–75 years	NHANES (1988–1994)	21 → 10
PCA	Zhang et al.	2014	China	505 People	35–91 years	Healthy population	114 → 7
PCA	Zhang et al.	2014	China	69 Males 70 Females	35–91 years	Healthy population	105 → 6
KDM	Belsky et al.	2015	New Zealand	954 People	38 years	The Dunedin Study (1972–1973)	10
KDM	Mitnitski et al.	2016	Canada	1,013 People (61.6% Females)	Average 80.8 ± 7.2 years	Canadian Study of Health and Aging (1991–1992)	22 → 10
DNN	Putin et al.	2016	Russia	62,419 People	0–100 years	Anonymous population	41
MLR/PCA/KDM	Jee and Park	2017	Korea	912 Females	30–80 years	Healthy population	31 → 8
PCA	Kang et al.	2017	Korea	165,395 Males 98,433 Females	Average 44.2 ± 10.6 years	Healthy population (including some early functional decline or disease)	5
PCA	Zhang et al.	2017	China	581 Males 792 Females	19–93 years	Healthy population	74 → 5
KDM	Brown et al.	2018	United States	1,356 Males 1,420 Females	70–79 years	The Health ABC Study (2013.11)	8
DNN	Mamoshina et al.	2018	Korea, Canada, Eastern Europe	142,379 People	≥20 years	Anonymous population	19
KDM	Murabito et al.	2018	United States	2,532–3,417 People	Average 45/62/67 years (Exam 2/7/8)	The Framingham Heart Study Exam 2 (1979–1983) Exam 7 (1998–2001) Exam 8 (2005–2008)	clinical BA:6 inflammatory BA:9
CNN	Pyrkov et al.	2018	United States	7,454 People (51% Females)	6–84 years	NHANES (2003–2006)	1-Week Activity Data
KDM	Hastings et al.	2019	United States	6,731 People (52% Males)	20–84 years	NHANES (1999–2002)	12
MLR/PCA/KDM	Jee	2019	Korea	940 Males	30–80 years	Healthy population	32 → 6
DNN	Mamoshina et al.	2019	Canada	149,000 People	Average 55 years	Anonymous population	18/20/23(three DNN models)
ConvLSTM	Rahman and Adjeroh	2019	United States	7,104 People	18–84 years	NHANES (2003–2006)	1-Week Activity Data
KDM	Gaydosh et al.	2020	China Taiwan	951 People	Average 67.7 ± 8.3 years	Social Environment and Biomarkers of Aging Study (2000)	11
KDM	Zuyun Liu et al.	2020	China	8,119 People (53.5% Females)	20–79 years	China Nutrition and Health Survey (2009)	27 → 12
KDM	Parker et al.	2020	United States	1,374 People (35% Males)	71–102 years	Duke Established Populations for Epidemiologic Studies of the Elderly (1991–1992)	10
MLR/PCA/KDM	Zhong et al.	2020	Singapore	2,844 People	55–94 years	Singapore Longitudinal Aging Studies (2008.03–2013.11)	68 → 8/10(Males/Females)
PCA/KDM	Chan et al.	2021	UK	141,254 People	40–70 years	Healthy population	110 → 51 principal components
DNN	Gialluisi et al.	2021	Italy	23,858 People (51.7% Females)	Average 55.9 ± 12.0 years	The Moli-Sani Study (2005.03–2010.04)	36
KDM	Kuo et al.	2021	UK	294,293 People	Average 56.7 ± 8.0 years	UK Biobank (2006–2010)	7
CNN	Raghu et al.	2021	United States	116,035 People	40–100 years	General population	Chest X-ray dataset
MLR/KDM	Bahour et al.	2022	United States	2,459 People	20–80 years	Diabetes, pre-diabetes, and NHANES (2017–2018) population	8
Deep learning	Nusinovici et al.	2022	Korea	40,480 People	≥65 years	Korean Health Screening study	retinal photos

MLR, multiple linear regression; PCA, principal component analysis; KDM, Klemera and Doubal’s method; DNN, deep neural networks; CNN, convolutional neural networks; ConvLSTM, deep convolutional long-term memory; NHANES, National Health and Nutrition Examination Survey.

## Selection of aging biomarkers

### Candidate biomarkers

The candidate biomarkers are a crucial factor to determine the final selected aging biomarkers. The most frequently used candidate biomarkers are routine clinical tests. These include age, sex, blood pressure, respiratory rate, pulse, heart rate, routine blood tests, blood biochemistry, routine urine tests, lung function, endocrine hormones (27, 34), and inflammatory factors (3, 38). Several metrics can be used to evaluate the same organ, and the best one is generally picked. For example, urea nitrogen (BUN), blood creatinine, and cystatin C (CYSC) are relevant to renal function. CYSC is a more sensitive marker of the endogenous glomerular filtration rate than blood creatinine (52). It has proven to be more suitable for BA models than blood creatinine and BUN (16, 30). Changes in body morphology (27, 29, 40) reflect the growth and nutritional statuses of the target population, such as waist circumference (WC), waist-to-hip ratio, waist-to-height ratio (WHtR), body mass index, and body fat. Abdominal obesity can occur in older adults with increased abdominal fat accumulation. Studies have shown that WHtR and WC are good indices to identify obesity in the elderly (53). Cognitive tests (4, 22, 45) are available to examine brain function, such as the trail making test, the digit symbol test, and the mini-mental state examination. Sensory tests (15, 29, 54), such as hearing, visual acuity, and vibration perception, are relatively less applied due to the cumbersome and specialist nature of the measurement process. Some parameters reflecting physical exercise capacities, such as grip strength (13) and vertical jump (31), are not only valuable for the BA model. They have also been used to structure physical (fitness) age (18, 19, 21) to assess aging. Imaging indices, such as cardiac and carotid ultrasound, are suitable biomarkers of aging for estimating BA (10, 16).

Aging is not a single process but is rather governed by a comprehensive range of factors, including disease, environment, lifestyle habits, and genetics. Health status, work experience, lifestyle, and dietary habits are often obtained through questionnaires (4, 22). Some genetic indicators are also taken as candidate aging biomarkers in the BA model, such as single nucleotide polymorphisms and terminal telomere restriction fragments (TRF). Nevertheless, probably because of its high detection cost in the population, genetic indicators are less applied in biological age. Zhang et al. (16) investigated polymorphic loci on P16, Sirt1, IL6, and Klotho genes associated with aging. Limited by the size of the sample population and the genes tested, the genes could not be used in BA models. TRF length is considered a genetic biomarker of aging at the cellular level (55), reflecting the dynamic aging process (56). TRF was found to be a promising aging biomarker in healthy aging populations (10).

### Selection criteria for aging biomarkers

Researchers or organizations in the field of aging have proposed criteria for selecting aging biomarkers, such as Butler et al. (57) and the American Federation for Aging Research (58), but no consensus has been reached. There are also some commonalities between the criteria, including aging biomarkers that predict age-related body functions, low or noninvasive assays, and high reproducibility (55). Costa and McCrae concluded that generic biomarkers of aging explain most of the changes that occur with increasing age (59, 60). This is similar to the criterion, proposed by Butler et al. (57), that aging biomarkers change with *CA.* An interesting question is if aging biomarkers can be selected according to their correlation with *CA.* First, it is associated with the method of estimating BA. When using MLR, PCA, and KDM methods, the initial step is to calculate the correlation between biomarkers and *CA.* This process is replaced by automatic machine learning in deep learning methods, which have a series of complex algorithms. Second, consider experiments that discovered biomarkers of aging and empirically found various physiological, biochemical, and imaging indicators that significantly correlate with age or analyzed age correlations from extensive data obtained from multiomics (2). In contrast, the selection of aging biomarkers in studies oriented to aging mechanisms is based on a hypothesis about the causes of aging (2). Finally, some investigators (61, 62) have given the more reasonable view that this correlation is not a reasonable criterion for selecting, validating, or weighting aging biomarkers, and some biomarkers moderately associated with CA may be utterly unrelated to aging. It follows that the correlation of aging biomarkers with CA is not equivalent to a causal relationship between aging biomarkers and senescence simply because there is no better alternative to correlation for screening markers. We summarized the standard selection criteria for aging biomarkers in the BA model (Table 2), including those significantly correlated with CA; nonredundant variables; monitoring the underlying mechanisms of the aging process rather than the effects of disease; repeatable measurements; reflecting different organ or physiological functions; and biomarkers used in previous studies.

**Table 2:** The selection criteria for aging biomarkers among MLR, PCA, and KDM.

Selection criteria	Assessment Methods
Significantly correlated with CA	MLR (3, 13, 14, 20–22, 27, 32, 35, 40, 45, 54), PCA (3, 4, 10, 16, 18, 19, 23–26, 28–30, 35, 40, 45, 54), KDM (3, 34, 35, 40, 45, 54)
Non-redundant variables	MLR (35, 40, 45), PCA (4, 10, 16, 25, 26, 28–31, 35, 36, 40, 45), KDM (35, 40, 43, 45)
Used in previous studies	MLR (3, 22, 27, 32, 54), PCA (3, 54), KDM (3, 33, 37–39, 42–44, 48, 54)
Monitors the underlying mechanisms of the aging process rather than the effects of disease	MLR (20, 32, 35, 40), PCA (29, 31, 35, 40), KDM (35, 40, 43)
Repeatable measurements	MLR (14, 35), PCA (35, 46), KDM (35, 38, 46)
Reflects different organs or physiological functions	MLR (13, 15, 54), PCA (15, 23, 54), KDM (38, 54)
Variables with higher loadings within the first principal component	MLR (3, 15), PCA (3, 10, 15), KDM (3)
Test results can be quantified	MLR (35), PCA (16, 30, 31, 35, 46), KDM (35, 46)

### Final selected aging biomarkers

It is critical to select the correct number of representative aging biomarkers to evaluate BA. The aging biomarkers of BA models in different systems are summarized in Table 3. In analyzing the differences in the final selection of aging biomarkers by different investigators, the preference is related to the study population. Some researchers (16, 18) choose all subjects to be healthy or almost healthy to exclude the effects of disease. There were also studies (31, 36) in which people in the early stages of adisease were included. In addition, the sample size, gender composition, age, and ethnicity of the study population can also impact the results. Moreover, BA models for specific populations, such as the elderly (37, 44) and the young (33), offer the possibility to explore aging differences between individuals within the same age group and potential clinical applications. Second, the choice is associated with candidate aging biomarkers; candidate biomarkers and investigators’ research directions and perceptions have a significant impact. Then, the choice of biomarkers is related to the selection criteria. The thresholds for correlation selection varied across trials. In recent years, some investigators (42, 44) building BA models referred to previously published biomarkers of aging, which are generally screened in large populations, used by others and have good reliability. Examples include the 10 biomarkers of aging that Levine initially selected in the third National Health and Nutrition Examination Survey (NHANES) population and the nine biomarkers of aging that were subsequently acquired through machine learning (3, 63). Finally, the selection is affected by the BA assessment approach. The MLR and PCA select aging biomarkers that correlate linearly with CA, and the KDM can be used in the nonlinear case of aging biomarkers (17). On the other hand, deep learning also answers whether candidate aging biomarkers can be selected through powerful fitting capabilities. One example is that chest radiographs, which researchers once discarded because they could not be quantified, have recently been used to construct BA models (49). Briefly, the final selection of aging biomarkers by research staff affected the study population, the candidate aging biomarkers, the selection criteria, and the method of BA evaluation.

**Table 3:** The common aging biomarkers of four methods in different systems.

System	MLR	PCA	KDM	Deep learning
Cardiovascular system	SBP (3, 13, 15, 20, 22, 27, 32, 35, 40)	SBP (3, 15, 18, 19, 23–26, 28, 29, 31, 35, 40)	SBP (3, 33, 35, 37–40, 42, 43, 48)	
	DBP (27, 32, 45)	DBP (45)	DBP (34, 45)	
	Pulse pressure (32)	Pulse pressure (4, 16, 30)		
		Mean arterial pressure (36)		
	Pulse (15)	Pulse (15, 18)		
	Pulse wave velocity (22)			
		Heart rate (19)		
		Intima-media thickness (10, 30)		
		Minimum intima-media thickness (4, 16)		
		End diastolic velocity (30)		
		mitral valve E/A peak (4, 30)		
		MVEL (30), MVES (16), MVEA (10)		
		Atherosclerosis index (18, 19)		
				NT-proBNP (47)
				Cardiac troponin I (47)
	Creatine phosphokinase (32)			
	Homocysteine (32)			
Respiratory system	FVC (15, 27, 32, 54)	FVC (15, 18, 19, 23, 24)	FVC (54)	
	FEV1 (3, 20, 21, 27, 32, 35, 40, 45)	FEV1 (3, 24–26, 28, 29, 31, 35, 40, 45)	FEV1 (3, 33, 35, 37, 38, 40, 45)	
	Vital capacity (13, 22)			
		Maximal midexpiratory flow rate 75/25 (16)		
		VO_2_ max (29, 31)		
				Chest radiography (49)
Nervous system	MMSE (45)	MMSE (45)	MMSE (45)	
	Digital symbol test (22)	Digital symbol test (10)		
	Numeric memory (54)		Numeric memory (54)	
	Associated memory (54)		Associated memory (54)	
	Topological memory (54)		Topological memory (54)	
	Short-time memory (14)			
	Concentration (54)		Concentration (54)	
	Intellectuality -mental defect (22)			
		Trail making test (4, 16)		
Endocrine metabolic system	Glucose (27, 32)	Glucose (23, 25, 36)	Glucose (38)	Glucose (8, 9, 41, 47)
	HBA1C (3, 32)	HBA1C (3, 29)	HBA1C (3, 33, 37, 39, 42–44, 48)	HBA1C (41)
				C-peptide (47)
				Insulin (47)
	Triglyceride (20, 27, 32)	Triglyceride (19, 36)	Triglyceride (43)	Triglyceride (9, 47)
	TC (3, 13, 15, 20, 21, 27, 32, 35)	TC (3, 15, 23, 35)	TC (3, 33, 35, 37, 38, 42, 43, 48)	TC (8, 9)
	HDL (27), LDL (32)	HDL (36), LDL (29)		HDL (9, 47),LDL (9, 47)
				Apolipoprotein A1 and B (47)
	TSH (27)		TSH (34)	
	Testosterone (27)			Testosterone (47)
	Vitamin D (40)	Vitamin D (40)	Vitamin D (40)	Vitamin D (47)
			Calcium (34)	Calcium (9)
				Potassium (9)
				Sodium (9)
			Inorganic phosphorus (34)	
Urinary system	Urea (3, 15, 20, 32, 35)	Urea (3, 15, 18, 19, 23, 24, 26, 28, 29, 35)	Urea (3, 33–35, 39, 42–44)	Urea (8, 9, 41)
	Creatinine (3, 27, 32, 35, 40)	Creatinine (35, 40)	Creatinine (3, 33, 34, 37, 39, 42–44, 48)	Creatinine (9, 47)
	eGFR (45)	eGFR (45)	eGFR (45)	
			Uric acid (39, 44)	Uric acid (47)
		Cystatin C (4, 10, 16, 30)		Cystatin C (47)
	Creatinine clearance (32)			
	Urine specific gravity (32)			
	Urine pH (32)			
Digestive system	ALT (32)	ALT (23)		ALT (47)
	AST (15, 32, 35)	AST (15, 18, 19, 35)	AST (35)	AST (47)
	ALP (3, 20, 32)	ALP (3)	ALP (3, 33, 34, 37, 39, 44, 48)	ALP (8)
	Total protein (32)		Total protein (34)	Total protein (9)
	Albumin (3, 15, 32)	Albumin (3, 15, 24, 26, 28, 29)	Albumin (3, 33, 34, 37, 39, 42–44, 48)	Albumin (8, 9, 47)
	A/G (15, 32)	A/G (15, 24, 25)		
	Total bilirubin (32)			Total bilirubin (9)
	Direct bilirubin (32)			
	Amylase (32)			
	Lactate dehydrogenase (21, 32)	Lactate dehydrogenase (19, 23)		
				Alpha 2 globulin (8)
	Gamma glutamyl transpeptidase (32)			
Hematologic System	Red blood cell (40)	Red blood cell (24, 28, 40)	Red blood cell (40, 43)	Red blood cell (8, 9, 47)
			Red blood cell volume distribution width (39)	Red blood cell volume distribution width (8, 47)
		Hematocrit (24, 26)		Hematocrit (8, 9)
			Mean corpuscular volume (39, 42, 44)	Mean corpuscular volume (9, 47)
		Mean corpuscular hemoglobin (25)		
				Mean corpuscular hemoglobin concentration (9, 47)
	Hemoglobin (15, 45)	Hemoglobin (15, 18, 19, 23, 24, 45)	Hemoglobin (34, 45)	Hemoglobin (9, 47)
			White blood cell (39, 42, 44)	White blood cell (47)
				Granulocytes (47)
				Neutrophils (47)
				Basophils (47), Eosinophils (47)
			Lymphocytes (39, 42, 44)	Lymphocytes (8, 47)
	Monocytes (45)	Monocytes (45)	Monocytes (45)	Monocytes (47)
			Platelet (43)	Platelet (9, 47)
				Mean platelet volume (47)
				Platelet distribution width (47)
	Erythrocyte	Erythrocyte		
	sedimentation rat (20, 27)	sedimentation rat (29)		
		D-dimer (10) Fibrinogen (30)		D-dimer (47)
	Ferritin (35)	Ferritin (35)	Ferritin (35, 43)	Ferritin (41)
			Fransferrin (43)	
Sensory system	Visual accommodation (22, 54)		Visual accommodation (54)	
	Visual reaction time (54)		Visual reaction time (54)	
	Visual acuity (13, 15)	Visual acuity (15)		
	Hearing (13, 21, 22, 54)	Hearing (29)	Hearing (54)	
	Vibrotactile (13, 14, 54)		Vibrotactile (54)	
				Retinal photos
Inflammatory index	CRP (3)	CRP (3)	CRP (3, 33, 37–39, 42–44, 48)	CRP (47)
	Cytomegalovirus optical density (3)	Cytomegalovirus optical density (3)	Cytomegalovirus optical density (3, 33, 42)	
			Interleukin-6 (38)	
			P-selectin (38)	
Motion index	Grip strength (13, 14, 45, 54)	Grip strength (31, 45)	Grip strength (45, 54)	
		Vertical jump (31)		
	Timed up and go test (45)	Timed up and go test (45)	Timed up and go test (45)	
	Chair rise time (45)	Chair rise time (45)	Chair rise time (45)	
				1-week physical activity (11, 12)
Body morphology index	WC (32, 35)	WC (29, 31, 35, 36)	WC (35)	
	Waist-to-hip ratio (27, 32)			
	Waist-to-height ratio (40)	Waist-to-height ratio (40)	Waist-to-height ratio (40)	
	Body mass index (27, 32)			
	Weight (22)			
	Height (45)	Height (45)	Height (45)	
	Body fat (27, 32)	Body fat (29)		
	Lean body mass (27, 32)			
		Soft lean mass (31)		
Genetic index		Terminal telomere restriction fragment (10)		
Genetic index		Terminal telomere restriction fragment (10)		

SBP, systolic blood pressure; DBP, diastolic blood pressure; NT-proBNP, N-terminal pro brain natriuretic peptide; MVEA, mitral annulus peak E anterior wall; MVEL, mitral valve annulus lateral wall of peak velocity of early filling; MVES, mitral valve annulus ventricular septum of the peak velocity of early filling; FEV1, forced expiratory volume in 1.0 s; FVC, forced vital capacity; MMSE, mini-mental state examination; eGFR, estimated glomerular filtration rat; HBA1C, glycosylated hemoglobin; HDL, high density lipoprotein; LDL, low density lipoprotein; TSH, thyroid stimulating hormone; ALT, Alanine aminotransferase; AST, Aspartate aminotransferase; ALP, alkaline *phosphatase; A/G, ratio of albumin to globulin; CRP, c-reactive protein; WC, waist circumference*.

## Biological age assessment method

### Multiple linear regression

Hollingsworth et al. (13) selected nine age-related indicators of physiological function and innovatively used MLR to predict BA in the Japanese Hiroshima population. This approach has since been widely used. The independent variable was selected according to the correlation between biomarkers and CA, and the individual’s BA was used as the dependent variable to establish the MLR equation:


(1) $$BA_{i}=b_{0}+\overset{m}{\underset{j=1}{\sum }}b_{j}x_{ji}$$

In Eq. (1), *m* is the number of aging biomarkers, and $x_{ji}$ (*i* = 1...*n*, *j* = 1...*m*) represents the *j*th aging biomarker of the *i*th individual (54). Moreover, *b_0_* and *b_j_* are the intercept and regression coefficients, respectively, calculated by the least-squares method. The BA model constructed using the MLR requires an F test for the significance of the regression equation, a t test for the significance of variables, and a goodness-of-fit test for how well the model fits the variables.

MLR has collinearity problems (54, 62), which can be diagnosed by the variance expansion factor method and characteristic root determination method, eliminating some unimportant independent variables and increasing the sample size to eliminate collinearity. MLR fails to avoid the biomarker paradox, where biomarkers perfectly associated with CA are insensitive in individuals (54). In addition, the BA values calculated by MLR are distorted at both ends of the regression equation (54, 62). Dubine et al. (14) proposed using the Z score to solve this problem. The equation for the Z score corrected BA equation is as follows:


(2) $$Corrected\;BA=BA_{i}+Z$$

Here, *Z* = (*CA_i_-MEAN_CA_*) × (*1-b*), where *CA_i_* is the chronological age of the individual, *MEAN_CA_* is the average chronological age of the population in which the individual is located, and *b* is the slope in the simple linear regression, representing the relationship between BA and *CA.*

### Principal component analysis

PCA was first used by Nakamura et al. (15) to estimate BA in Japanese populations, subsequently becoming popular in Korea, China, and other countries (3, 16, 29, 30). Our team was the first to utilize PCA to evaluate BA in healthy populations in China (30). For nearly 20 years, we have structured BA models based on data from single-centered and multicentered populations, finding that aging biomarkers such as CYSC and carotid intima-media thickness were closely associated with aging in the Chinese population (4, 10, 16, 30).

The steps of building a BA model by PCA are summarized as follows: (1) select aging biomarkers by correlation analysis, stability analysis, and redundancy analysis (64); (2) convert potentially relevant aging biomarkers into linearly uncorrelated principal components by orthogonal transformation; and (3) select the first principal component or multiple principal components to create the formula for predicting BA. When the PCA selects only the first principal component, the critical aging biomarkers can be further screened by principal component loading (3). The colinearity problem can be avoided if multiple principal components are selected as new variables (54). Both approaches have their advantages. The biological age score (BAS) formula constructed by selecting the first principal component as an example is as follows:


(3) $$BAS=a_{1}X_{1}^{\prime }+a_{2}X_{2}^{\prime }+\ldots +a_{n}X_{n}^{\prime }$$

Here, *X’* is the standardized biomarker of aging, *a_n_* is the score coefficient of aging biomarkers, where *X’* is computed from the equation $\frac{X-MEAN\left(X\right)}{SD\left(X\right)}$ and *MEAN(X)* and *SD(X)* are the mean and standard deviation of the aging biomarkers, respectively. Because the BAS is not measured in years, Nakamura et al. (15) used the T-score to convert BAS to BA:


(4) $$BA=BAS\times SD_{CA}+MEAN_{CA}$$

In Equation (4), *SD_CA_* and *MEAN_CA_* are the variance and mean of the chronological age of subjects, respectively. Similar to the MLR method, to avoid regression of BA values toward the mean age of the sample, the Z score was used to correct BA (10, 16), as in Formula (2).

### Klemera and Doubal’s method

KDM was first proposed by Klemera and Doubal (17) in 2006 and is now broadly available for aging and aging-related research. The KDM formula development process incorporates certain core hypotheses (17): (1) The hypothesis is that “the difference in BA is the difference among individuals aging in the same CA population.” A random variable $R_{BA}\left(0;S_{BA}^{2}\right)$ with mean 0 and variance $S_{BA}^{2}$ was used to replace the differences in individual BA, establishing the formula:


(5) $$BA=CA+R_{BA}\left(0;S_{BA}^{2}\right)$$

(2) Another hypothesis is that “the actual value of aging biomarker X is not only regulated by BA but also influenced by transient effects that are not BA dependent” (17). A random variable $R_{X}\left(0;S_{X}^{2}\right)$ with mean 0 and variance $S_{X}^{2}$ is used to represent the transient effect. An inverse regression equation similar to the Hochschild (65) view is obtained:


(6) $$X=F_{X}\left(BA\right)+R_{X}\left(0;S_{X}^{2}\right)$$

Here,$\;F_{X}\left(BA\right)$ is considered a simple linear equation with independent variable BA, intercept q, and slope k. Subsequently, Klemera and Doubal developed two formulations, shown in Eqs (7) and (8), for calculating BA through sophisticated mathematical derivations, the distinction being the inclusion of CA as an independent variable in Eq. (8) (17).


(7) $$BA_{E}=\frac{\sum_{j=1}^{m}\left(x_{j}-q_{j}\right)\left(\frac{k_{j}}{s_{j}^{2}}\right)}{\sum_{j=1}^{m}{\left(\frac{k_{j}}{s_{j}}\right)}^{2}}$$

(8) $$BA_{EC}=\frac{\sum_{j=1}^{m}\left(x_{j}-q_{j}\right)\left(\frac{k_{j}}{s_{j}^{2}}\right)+\frac{CA}{S_{BA}^{2}}}{\sum_{j=1}^{m}{\left(\frac{k_{j}}{s_{j}}\right)}^{2}+\frac{1}{S_{BA}^{2}}}$$

where $x_{j}$, $q_{j}$, $k_{j}$, and $s_{j}^{2}$ (*j* = 1...*m*) represent the *jth* aging biomarker and its intercept, slope, and transient effect, respectively. The detailed derivation process is available in their paper. Cho et al. (54) improved the algorithm of KDM to simplify the computing flow. In the KDM2 model they developed, PCA was introduced, and multiple sets of principal components were selected instead of the original aging biomarkers (54). Levine (3) used the modified method that combined PCA to construct the KDM2 model, with the difference being that they selected the key aging biomarkers within the first principal component. In addition, the Δage (Δage = BA-CA) was determined by the KDM method and is more practical than calculating the BA of an individual (34). Recently Kwon and Belsky developed an R package containing the KDM method: BioAge, for facilitating biological age measurement (66).

### Deep learning

Deep learning is a subfield of machine learning, where good features can be learned automatically using a general-purpose learning procedure (67). Deep neural networks (DNNs) (8, 9, 41, 47), convolutional neural networks (CNNs) (11, 49), and recurrent neural networks (RNNs) (12) have been employed to build BA models in recent years.

A DNN consists of an input layer that receives external data, several hidden layers responsible for feature extraction, and an output layer that outputs the final result. Round-by-round iterations are performed with activation functions (68), gradient descent (69), and backpropagation algorithms to transform the input data into results for solving regression or classification problems. In 2016, Putin et al. constructed a model for predicting BA using a DNN, which is placed at www.aging.ai for public usage; the BA can be estimated by entering the complete 41 blood markers or just the most crucial 10 markers (8). The model performed poorly in non-Eastern European populations due to population differences (70). Mamoshina et al. trained a DNN with a dataset containing ethnically diverse populations to enhance accuracy in computing BA in Canadian, Korean, and Eastern European populations (9). More recently, Gialluisi et al. created a DNN based on 36 circulating biomarkers to estimate BA in Italians (47). It is worth considering that suitable approaches should be explored in deep learning to clarify the weight and importance ranking of individual aging biomarkers to assess their importance. Permutation feature importance (PFI) is an algorithm derived from the random forest that explains the importance of aging biomarkers in DNNs (8, 9). To explore the effect of smoking on BA, Mamoshina et al. used PFI to rank biomarkers and selected the top-ranked biomarkers to construct three DNNs and found that smoking accelerates aging (41).

Used chiefly for image analysis, CNNs are mainly composed of convolutional and pooling layers with features such as local connectivity and weight sharing, simplifying the number of parameters and the complexity of calculations (67). The convolutional layer converts the input information into an output feature map with multiple feature mappings employing a specific number of filters. The pooling layer is intended to reduce the information output from the convolution layer. It is executed immediately after the convolution, completed by the maximum, minimum, or average value of operations. Pyrkov et al. selected physical activity data recorded by wearable devices of NHANES participants for 1 week as a biomarker of aging and generated a BA model with a CNN (11). Raghu et al. built a BA model using chest radiograph data combined with a CNN (49).

RNNs are designed with a loop/repeat structure to preserve valuable historical information in sequences through “state vectors” to better process sequence data. RNNs suffer from long-term dependency problems (71) such as vanishing or exploding gradient when learning on long sequences. Hochreiter and Schmidhuber (72) proposed the long short-term memory network (LSTM) to solve this problem, adding the *cell state* unit and the *gate unit* based on the ordinary RNN. Thus, LSTM can handle short-term information sensitively and remember valuable information for a long time, which improves the network’s learning ability. Rahman and Adjeroh (12) also used physical activity data from 1 week. Considering the temporal information contained in the data, they adopted a deep convolutional long-term memory (ConvLSTM) method to develop a BA model that outperformed other deep learning approaches (12).

### Other methods

In addition to the four popular methods mentioned above, some researchers have tried to find the best model to assess BA, starting from the aspect of the impact of aging markers on life expectancy. Hochschild (62, 65, 73) suggested a nonstandard, complex, but reasonable approach to estimating BA. Taking a questionnaire format, he collected mortality risk factors, such as smoking, diet, and exercise, and aggregated these indicators into a “composite validation variable (CVV).” The standardized biological age was then calculated by the correlation coefficient between CVV and aging markers and finally transformed into BA in years. Some studies used correlations with mortality to identify aging variables. Twelve clinical indicators associated with mortality were selected by Drewelies et al. and validated in two independent birth cohorts (74). Levin and coworkers (63, 75) determined the “phenotypic age” through a multifactorial analysis of mortality risk, an algorithm known to some researchers (39, 44) as “LM BA.” First, nine aging biomarkers associated with mortality were selected with machine learning. Then, two Gompertz proportional hazards models were developed to predict the mortality risk, called the “mortality score,” and converted to biological age values. In addition, Pyrkov et al. constructed BA models to predict both CA and life expectancy, further discussing the relative performance of the models in stratifying the effects of diseases and lifestyles (11). However, these approaches are not widely used for two main reasons: one, a mortality event is required to calculate the aging rate, which is a lengthy process for the follow-up of a normally aging population; second, even if death occurs, many cannot distinguish whether it is due to death from disease or natural death from aging.

#### Comparison of the four assessment methods

There is no perfect way to evaluate BA. Each researcher should select the proper approach according to the study’s purpose, sample population, laboratory conditions, funding, statistical knowledge, and programming ability. A summary of the strengths, limitations, and possible practical improvements of the four BA assessment methods is presented in Table 4. MLR is the simplest way to measure BA but suffers from the biomarker paradox, edge distortion, and colinearity (54). “collinearity” refers to linear regression in which the accuracy of the model may be affected by the presence of highly correlated relationships among the independent variables, and “edge distortion” refers to distortion at both ends of the linear regression, where the estimated BA values are too large or too small. PCA can further screen aging biomarkers or solve the covariance problem. However, PCA selects aging markers based on correlation with CA and cannot settle the paradox of biomarkers. KDM uses a reverse regression equation that solves the biological paradox (65) and avoids distortion at both ends of the regression equation (76). The KDM2 model combined with PCA allows for a better valuation of mortality (3). Although *BA_EC_* is superior to *BA_E_* in predicting BA, assigning CA a similar role to other biomarkers would be controversial, as proposed by Klemera and Doubal (17). Mitnitski et al. (34) argued that CA is made an independent variable to avoid extreme values (to prevent BA from being overcalculated, e.g., 120 years) at the expense of BA clarity. Deep learning allows machines to extract features and construct BA models autonomously by learning about biomarkers. Their strengths include the ability to handle high-dimensional datasets with complex interactions and correlations to resolve problems that people do not fully understand. However, it is challenging to structure large datasets (8), and there is a “black box” in deep learning, where the specific learning process is unknown, and the results are uncontrollable.

**Table 4:** The advantages and disadvantages of the four methods and possible effective improvements.

Methods	Advantages	Disadvantages	Improvements
MLR	Simple and easy to operate	(1) Biomarkers paradox (2) co-linearity (3) Regression equation edge distortion	(1) Z-score correction edge distortion (14) (2) Co-linearity diagnosis and removal of redundant variables
PCA	(1) Avoid co-linearity (54) (2) Further screen aging biomarkers (3)	(1) Biomarkers paradox (2) Regression equation edge distortion	Z-score correction edge distortion (14)
KDM	(1) Resolving the biomarker paradox (2) Avoiding distortion at the edges of the regression equation (76) (3) Suitable for non-linear biomarkers (17)	(1) Complicated calculation (54) (2) CA as a marker of aging is controversial (34)	(1) Cho et al. improved the calculation process (54) (2) Calculating individual △age is more practical than BA (34)
Deep learning	(1) Good at handling high-dimensional dataset (67) (2) The machine extracts features autonomously by learning (67)	(1) Difficulty in building large data (8) (2) The existence of a “black box” and uncontrollable results (3) Excellent programming skills and computer hardware and software support required	(1) Suitable methods can be explored to clarify the weighting of each aging biomarker (2) Multidisciplinary Cooperation

MLR, multiple linear regression; PCA, principal component analysis; KDM, Klemera and Doubal’s method.

#### Performance evaluation of biological age

Currently constructed BA models are mainly used to Predict the occurrence of disease or predict life expectancy. Their performance evaluation metrics, such as R (2), reflect the superiority of the training model by the degree of fit between the estimated BA and the actual CA, however, the nonlinear fitting strategy in machine learning can cause *R*^2^ to suffer from overfitting problems. In contrast, Δage is superior, which response to the acceleration of biological age. The validity of the model was recently confirmed by Li and Zhang et al. who trained the BA model using a healthy Beijing population and later validated the acceleration of aging by disease in a diseased population (77). However, the value of ∆age may only serve a qualitative purpose, as relative acceleration or retardation of aging compared to a healthy population with the same *CA.* Some researchers have checked the efficacy of BA models through mortality (78). The introduction of a BA model with mortality training is similar to the inclusion of endpoint events in clinical trials. On the one hand, the incorporated life-length data may improve the accuracy of BA models more than the iteration of statistical methods alone; on the other hand, the exploration of endpoint mortality events may be more likely to generate public interest in BA. However, some emergencies or sudden events may affect the accuracy of biological age judgment.

## Summary and prospects

BA is an integral part of the aging field. Only by accurately evaluating the individualized aging status, prompting an earlier window of aging prevention, and timely intervention for disease-prone and diseased individuals can we improve the quality of life of the elderly and prolong their lifespan (79). We investigated the selection and assessment methods of aging biomarkers in BA. Although researchers in aging have identified some aging biomarkers, due to the continuous application of new technologies, new ideas, and new groups of people, there are still many potentially better biomarkers waiting to be discovered. For example, with the increasing availability of longitudinal biological data, the organic state recovery time (resilience) has been found to be an important marker of aging, which cannot be obtained from cross-sectional data (80, 81). Moreover, the development of sequencing omics and image acquisition has provided a direction for exploring new aging biomarkers.

This review focuses on statistical methods for quantifying BA, discusses the advantages and disadvantages of different statistical methods, summarizes the different kinds of aging markers applied in biological age, also mentioned the selection criteria of aging markers. It compares traditional methods and new deep learning methods in BA research and helps clarify how to construct a more accurate new biological age. Of course, this paper has the following shortcomings: on the one hand, candidate markers and selected markers are different for each article, it cannot reflect the importance of each marker by the frequency of aging markers use; on the other hand, only a brief discussion on the performance aspects of statistical methods is made in this paper, and longitudinal or mortality verification is less; Third, there are few studies using the same population to compare the pros and cons of different methods, and more are theoretically compared. We suggest that, regarding the selection of aging markers, it is necessary to focus on both the discovery of new aging markers and the reassessment of the role of old aging markers. It is very important to develop standardized or equivalent screening criteria for aging markers. The new method is more accurate but requires a larger amount of data, and it is easy to overfit if the amount of data is small, so it is not the latest that is the best, but the right one is the best. Common BA evaluation methods have their own merits and demerits and are continuously optimized in practice. The combined use of multiple methods may yield superior results and facilitate the creation of new methods. Biological age is mostly constructed in healthy or community populations to avoid the influence of disease factors. We should pay more attention to the accuracy of applying biological age in different populations such as disease populations, progeria populations, and longevity populations. In the future, we should pay attention to the clinical translation of BA models and do further exploration for clinical utility, such as developing assessment models suitable for different disease individuals and further revealing the complex interaction between disease and aging.

## Author contributions

ZL and WZ was written the first draft of the manuscript. YD, YN, YC, XL, ZD, YZ, XzC, ZF, YW, and DZ commented on previous versions of the manuscript. XS, GC, HJ, and XmC provided enough scientific suggestions and concrete actions during the revision. All authors read and approved the final version of the manuscript.

## Funding

This study was supported by National Key Research and Development Program of China (2022YFC3602900, 2022YFC3602903, 2022YFC3602902), Innovation Platform for Academicians of Hainan Province (Academician Chen Xiangmei of Hainan Province Kidney Diseases Team Innovation Center), the Specialized Scientific Program of the Innovation Platform for Academicians of Hainan Province (YSPTZX202026), the Specialized Scientific Research Project of Military Health Care (21BJZ37), the Clinical Research Support Fund, Young Talent Project, Chinese PLA General Hospital (2019XXMBD-005, 2019XXJSYX01), the National Natural Science Foundation of China (82030025, 81601211).

## Conflict of interest

The authors declare that the research was conducted in the absence of any commercial or financial relationships that could be construed as a potential conflict of interest.

## Publisher’s note

All claims expressed in this article are solely those of the authors and do not necessarily represent those of their affiliated organizations, or those of the publisher, the editors and the reviewers. Any product that may be evaluated in this article, or claim that may be made by its manufacturer, is not guaranteed or endorsed by the publisher.
